# In Memoriam—Dr. Grey A. T. Lincoln

**Published:** 1895-11

**Authors:** 


### IN MEMORIAM—DR. GREY A. T. LINCOLN.

At the September meeting of the Bœnninghausen Club a committee was appointed to draft resolutions upon the death of Dr. Lincoln, as follows:

WHEREAS, God in His Providence has suddenly removed from our midst one of our members, Grey A. T. Lincoln, M.D., one whose familiar face and form we were wont to see at our meetings, and whose voice was often heard in discussing those questiont so dear to his heart as a follower of Hahnemann; therefore,

*Resolved*, That in the death of our colleague, we have lost one who was a true friend, a kind son, and a faithful adherent to the teachings of Homœopathy.

*Resolved*, That a copy of these resolutions be printed in the forthcoming number of THE HOMŒOPATHIC PHYSICIAN and that a copy be sent also to the family of the deceased.

A. L. KENNEDY,
R. L. THORNTON,
W. P. DEFRIEZ,
*Committee.*

Dr. Lincoln died suddenly at his home at 769 Tremont Street, Boston, on the 29th day of July.

As a medical student he received a wound while dissecting, which troubled him somewhat, though not seriously at the time. He frequently said that he had "never been in robust health since." Latterly he complained of severe headaches from time to time. He prophesied that he should die suddenly of apoplexy, and thus it proved. He attended to his patients as usual the day before his death.

He was a firm believer in Homœopathy, and a conscientious practitioner of the principles promulgated by Hahnemann. We can ill afford to lose such men from our ranks.

